# Genome mapping and expression analyses of human intronic noncoding RNAs reveal tissue-specific patterns and enrichment in genes related to regulation of transcription

**DOI:** 10.1186/gb-2007-8-3-r43

**Published:** 2007-03-26

**Authors:** Helder I Nakaya, Paulo P Amaral, Rodrigo Louro, André Lopes, Angela A Fachel, Yuri B Moreira, Tarik A El-Jundi, Aline M da Silva, Eduardo M Reis, Sergio Verjovski-Almeida

**Affiliations:** 1 Departamento de Bioquimica, Instituto de Quimica, Universidade de São Paulo, 05508-900 São Paulo, SP, Brazil

## Abstract

**Background:**

RNAs transcribed from intronic regions of genes are involved in a number of processes related to post-transcriptional control of gene expression. However, the complement of human genes in which introns are transcribed, and the number of intronic transcriptional units and their tissue expression patterns are not known.

**Results:**

A survey of mRNA and EST public databases revealed more than 55,000 totally intronic noncoding (TIN) RNAs transcribed from the introns of 74% of all unique RefSeq genes. Guided by this information, we designed an oligoarray platform containing sense and antisense probes for each of 7,135 randomly selected TIN transcripts plus the corresponding protein-coding genes. We identified exonic and intronic tissue-specific expression signatures for human liver, prostate and kidney. The most highly expressed antisense TIN RNAs were transcribed from introns of protein-coding genes significantly enriched (*p *= 0.002 to 0.022) in the 'Regulation of transcription' Gene Ontology category. RNA polymerase II inhibition resulted in increased expression of a fraction of intronic RNAs in cell cultures, suggesting that other RNA polymerases may be involved in their biosynthesis. Members of a subset of intronic and protein-coding signatures transcribed from the same genomic loci have correlated expression patterns, suggesting that intronic RNAs regulate the abundance or the pattern of exon usage in protein-coding messages.

**Conclusion:**

We have identified diverse intronic RNA expression patterns, pointing to distinct regulatory roles. This gene-oriented approach, using a combined intron-exon oligoarray, should permit further comparative analysis of intronic transcription under various physiological and pathological conditions, thus advancing current knowledge about the biological functions of these noncoding RNAs.

## Abstract 2

An analysis of the expression of 7,135 human totally intronic noncoding RNA transcripts plus the corresponding protein-coding genes using oligonucleotide arrays has identified diverse intronic RNA expression patterns, pointing to distinct regulatory roles.

## Background

The five million expressed sequence tags (ESTs) deposited into public sequence databases probably constitute the best representation of the human transcriptome. Human EST data have been extensively used to identify novel genes *in silico *[1,2] and novel exons of protein-coding genes [3-6]. Informatics analyses of the EST collection mapped to the human genome have also shown that the occurrence of overlapping sense/antisense transcription is widespread [7-9]. However, the complement of unspliced human transcripts that map exclusively to introns was not appreciated in those reports because the authors selected: transcripts with evidence of splicing [7]; pairs of sense-antisense messages for which at least one exon was colinear on the genome sequence [8]; or only ESTs where both a polyadenylation signal and a poly(A) tail were present [9].

A detailed analysis of the mouse transcriptome based on functional annotation of 60,770 full-length cDNAs revealed that 15,815 are noncoding RNAs (ncRNAs), of which 71% are unspliced/single exon, indicating that ncRNA is a major component of the transcriptome [10]. The recent completion and detailed annotation of the euchromatic sequence of the human genome has identified 20,000 to 25,000 protein-coding genes [11]; however, noncoding messages were not assessed [11]. Extrapolation from the numbers for chromosome 7 leads to an estimate of 3,700 human ncRNAs [12], and two databases of human and murine noncoding RNAs are available [13,14]. Nevertheless, there has been no comprehensive count and mapping of human noncoding RNAs.

Examples of long (0.6-2 kb) intronic noncoding RNAs involved in different biological processes are described in the literature; they participate in the transcriptional or post-transcriptional control of gene expression [15,16], and in the regulation of exon-skipping [17] and intron retention [18]. In addition, microarray experiments performed by our group have revealed a set of long intronic ncRNAs whose expression is correlated to the degree of malignancy in prostate cancer [19]. Introns are also the sources of short ncRNAs that have been characterized as microRNAs [20] and small nucleolar RNAs (snoRNAs) [21]. Biogenesis and function are better understood for microRNAs than for other ncRNAs; they may regulate as many as one-third of human genes [20], and tissue-specific expression signatures have been identified in different human cancers [22]. However, the complement and biological functions of most of the complex and diverse ncRNA output, both the short and the long ncRNAs, remain to be determined.

Different types of noncoding RNA genes can be transcribed by either RNA polymerase (RNAP) I, II or III [15]. Recently, a fourth nuclear RNAP consisting of an isoform of the human single-polypeptide mitochondrial RNAP, named spRNAP IV, was found to transcribe a small fraction of mRNAs in human cells [23]. Surprisingly, α-amanitin up-regulates the transcription of protein-coding mRNAs by this polymerase [23]. The role of spRNAP IV in the transcription of ncRNAs has not been investigated.

Here we report a search for hitherto unidentified exclusively intronic unspliced RNA transcripts in the collection of transcribed human sequences available at GenBank. The characterization comprises the identification and distribution analysis of 55,000 long intronic ncRNAs over the introns of protein-coding genes and the detection of a higher frequency of alternatively spliced exons for genes that undergo intronic transcription. An oligoarray with 44,000 elements representing exons of protein-coding genes and the corresponding actively transcribed introns was employed to assess intronic transcription in different human tissues. Robust tissue signatures of exonic and intronic expression were detected in human kidney, prostate and liver. We found that in each tissue, the most highly expressed exclusively intronic antisense RNAs were transcribed from a group of protein-coding genes that is significantly enriched in the 'Regulation of transcription' Gene Ontology (GO) category. A subset of partially intronic antisense ncRNAs and the corresponding overlapping protein-coding exons showed a correlated pattern of tissue expression, indicating that intronic RNAs may have a role in regulating abundance or alternative exon-splicing events. Finally, we found that a significant fraction of wholly or partially intronic ncRNAs is insensitive to RNAP II inhibition by α-amanitin, and another fraction is even up-regulated when RNAP II transcription is blocked, suggesting that a portion of long ncRNAs may be transcribed by spRNAP IV. We conclude that oligoarray-based gene-oriented analysis of intronic transcription is a powerful tool for identifying novel potentially functional noncoding RNAs.

## Results

### Defining a comprehensive reference dataset of spliced protein-coding genes

To analyze the complex distribution of transcriptionally active regions on a genome-wide scale, we started by mapping the set of well-annotated 22,458 RefSeq transcripts to the human genome sequence. We excluded 1,184 unspliced RefSeq and 601 RefSeq that were wholly intronic to another RefSeq. When the spliced RefSeq transcripts mapping to the same locus were merged, we identified a set of 15,783 non-redundant spliced RefSeq units. Thus, a total of 4,890 RefSeq representing isoforms of the same genes were merged into these units. In addition, the GenBank mRNA sequence dataset was mapped to the genome in order to document splice variants present in that set but not in the non-redundant RefSeq data. For this purpose, 161,993 human mRNAs from GenBank were mapped to the human genome, as described in Materials and methods. Initially, they were clustered into a total of 45,137 transcriptional units mapping to unique loci in the genome (Table 1).

**Table 1:** Evidence of intronic transcription in the human mRNA/RefSeq GenBank dataset

	mRNA clusters with overlap to exons of non-redundant RefSeq dataset*		mRNA clusters wholly intronic to non-redundant RefSeq dataset			
				
	Antisense direction	Sense direction	Antisense direction	Sense direction	mRNA clusters not mapped to RefSeq dataset	Total
Spliced mRNA clusters^†^	2,559 (1,414)^‡^	14,575 (14,369^§^)	1,049 (378)	780 (223)	4,181 (0)	23,144 (16,384)
Unspliced mRNA clusters^†^	1,672 (26)	7,463 (87)	1,456 (56)	4,222 (87)	7,180 (927)	21,993 (1,183)
Total	4,231 (1,440)	22,038 (14,456)	2,505 (434)	5,002 (310)	11,361 (927)	45,137 (17,567)

*The non-redundant dataset comprises 15,783 spliced RefSeq units. This was defined by mapping to the human genome sequence the total of 22,458 RefSeq sequences from GenBank, excluding 1,184 unspliced RefSeq and 601 RefSeq that were wholly intronic to another RefSeq and merging the remaining 20,673 spliced RefSeq sequences that mapped to the same locus into 15,783 spliced non-redundant RefSeq units (a total of 4,890 RefSeq that represent isoforms of the same gene were thus merged into these units). ^†^mRNA clusters were obtained by mapping to the human genome sequence a total of 161,993 mRNA sequences followed by merging sequences with exon overlapping coordinates (see Materials and methods for details), resulting in a non-redundant set of 45,137 mRNA clusters. This set was aligned to the non-redundant RefSeq dataset and each mRNA cluster was classified as exonic, wholly intronic or mapping outside of any spliced non-redundant RefSeq unit. Sense/antisense orientation was annotated. ^‡^For each class, the number of mRNA clusters containing at least one RefSeq is shown in parentheses. ^§^Excluding from the 15,783 spliced non-redundant RefSeq dataset a total of 1,414 RefSeq that map in the antisense direction with respect to another RefSeq.

A detailed analysis of the mapping coordinates of these mRNA clusters with respect to the non-redundant RefSeq dataset revealed that 11,361 spliced and unspliced clusters mapped outside the non-redundant RefSeq dataset, representing less well-characterized human transcripts. As expected, most of the mRNA clusters (14,575) were spliced and mapped to exons of RefSeq genes in the sense direction (Table 1). In addition, 2,559 spliced mRNA clusters mapped in the antisense direction with respect to the non-redundant RefSeq dataset, suggesting that 16% of the RefSeq genes have spliced natural antisense transcripts that overlap at least one of their exons. Among these antisense messages, 1,414 are already annotated as RefSeq transcripts. Such genomic organization of sense-antisense gene pairs seems to have been conserved throughout vertebrate evolution [7,8,24,25]. When the unspliced mRNA clusters were included, we found a total of 4,231 antisense messages with overlaps to exons in RefSeq genes, indicating that as many as 27% of the latter have antisense counterparts. A complete list of these sense/antisense pairs with exon overlapping is given in Additional data file 1. This is in line with the prediction that over 20% of human transcripts might form sense-antisense pairs [9]. As a control, we cross-referenced the previously known sense/antisense pairs to our dataset (see Materials and methods) and found that essentially 100% of known pairs [8,9] with evidence from RefSeq or mRNA are covered by our set. In addition, we found 1,116 RefSeqs with evidence of antisense exon-overlapping messages not covered by Yelin *et al*. [8] and 1,573 not covered by Chen *et al*. [9]. The complete list of sense/antisense pairs identified here is given in Additional data file 1 along with data for the cross-reference to published sense/antisense pairs.

Most interestingly, we found 7,507 spliced and unspliced mRNA clusters that are entirely intronic to the non-redundant RefSeq genes (Table 1). While 5,002 (67%) of these mapped in the sense direction and may represent new exons of the corresponding genes, 2,505 (33%) mapped exclusively to the introns of RefSeq genes in the antisense direction and thus comprise a set of antisense mRNA clusters with no overlap to exons of sense messages that had not been appreciated in the previous analyses. A complete list of the latter wholly intronic mRNA/RefSeq clusters and the corresponding protein-coding RefSeq is given in Additional data file 1. Although the strandedness of genomic mapping of these mRNAs was taken as preliminary evidence of antisense transcription, direct experimental confirmation was obtained by microarray assays, as described in the following sections. Owing to the fragmented nature of the transcript data in GenBank, some of these intronic antisense messages may originate from the 3' or 5' ends of overlapping sense-antisense transcripts of adjacent genes. However, most of them could represent independent antisense transcriptional units, which became more evident when data from the public EST repository were taken into account, as described below.

### Identification of long, unspliced, totally intronic transcripts

**Figure 1:**
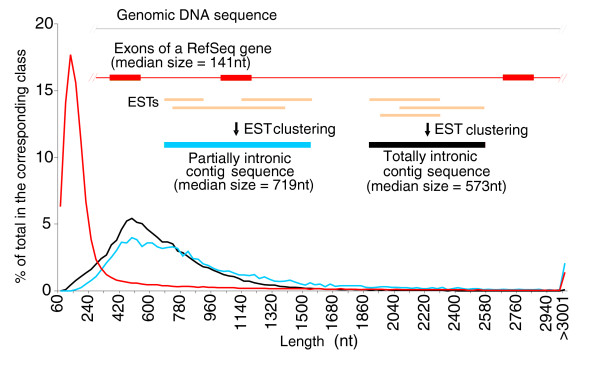
Length distribution of exons from RefSeq genes and of partially (PIN) and totally (TIN) intronic noncoding transcripts. The curves show the length distribution of three different classes of transcripts reconstructed from genomic mapping and assembly of RefSeq and ESTs from GenBank. Exons of protein-coding RefSeq (red line), TIN (black line) and PIN (blue line) contig sequences. TIN and PIN contigs resulted from assembly of all GenBank unspliced ESTs (in gold) that cluster to a given intronic region in a genomic locus, as shown in the scheme above the curves.

We performed an extensive search for evidence of intronic transcription in the human dbEST collection (GenBank) comprising 5,340,464 ESTs. Ambiguously mapping EST sequences were filtered as described in Materials and methods, and then the genomic coordinates of overlapping EST sequences were used to merge 4,762,523 human ESTs into a set of 332,946 non-redundant EST clusters (Table 2). To avoid sequences that may have been derived from genomic contamination in the EST dataset, 210,181 EST singlets were excluded from further analyses; so only 34,398 spliced and 88,367 unspliced EST clusters were considered (Table 2). For each of these clusters, a consensus contig sequence was derived from the aligned genomic sequence (Figure 1). As expected, most ESTs (3,616,644) were grouped into 16,241 spliced EST contigs mapping to exons of the RefSeq reference dataset (Table 2). In addition, a small number of spliced EST clusters mapped to introns of the RefSeq genes. They may constitute fragments of novel exons in these genes, since the median exon length in these spliced EST contigs is 233 nucleotides (nt), similar to the median length of exons in the RefSeq reference dataset (141 nt).

**Table 2:** Classification of GenBank ESTs with respect to their genome mapping coordinates in relation to the set of non-redundant spliced RefSeq sequences

	EST clusters with overlap to exons of RefSeq genes*	EST clusters wholly intronic to RefSeq genes	EST clusters mapped outside of RefSeq genes	Total
Spliced EST contigs	16,241	8,013	10,144	34,398
Number of exons of spliced EST contigs (median)	10	2	3	
Total number of spliced ESTs in contigs	3,616,644	162,841	241,049	4,020,534
Number of spliced ESTs per contig (median)	91	3	4	
Unspliced EST contigs	4,030	55,139	29,198	88,367
Total number of unspliced ESTs in contigs	56,752	190,583	140,091	387,426
Number of unspliced ESTs per contig (median)	4	2	2	
Spliced EST singlets	1,053	6,205	6,631	13,889
Unspliced EST singlets	3,539	121,091	71,662	196,292
Total non-redundant EST clusters (contigs + singlets)	24,863	190,448	117,635	332,946
Total ESTs	3,677,988	480,720	459,433	4,618,141

*The reference dataset comprises 15,783 spliced non-redundant RefSeq units plus the evidence of additional splice variants obtained for each transcriptional unit from all mRNA sequences mapping to the same locus.

The most interesting finding was that 55,139 unspliced EST contigs formed by grouping 190,583 ESTs mapped entirely to the introns of genes in the RefSeq dataset (Table 2). A marked feature of these unspliced, wholly intronic EST contigs is their low protein-coding potential; *in silico *analysis of the coding potential using the normalized ESTScan2 score [26] predicted that 98% of them are probably noncoding transcripts, supporting the idea that they represent a separate class of noncoding RNAs. To check whether ESTScan2 predicted the coding potential of such a fragmented sequence dataset correctly, we created a virtual dataset *in silico *composed of 55,139 exonic fragments from RefSeq genes with exactly the same lengths as the 55,139 wholly intronic EST contigs. ESTScan2 correctly predicted that 70% of these *in silico*-generated virtual exonic fragments have coding potential. This supports the inference that since only a very few (approximately 2%) of the wholly intronic EST contigs are predicted by ESTScan2 to have a protein-coding potential, most of the RNAs in this class (98%) are indeed noncoding messages.

Inspection of the length distribution curves (Figure 1) of the wholly intronic EST contigs reveals messages with lengths well over 1,000 nt. The median length (573 nt) is 4.1 times greater than the median length of exons (141 nt) in the RefSeq reference dataset. On the basis of these findings, we call these transcriptional units long totally intronic noncoding (TIN) transcripts.

Most mammalian snoRNAs [21] and a large fraction of microRNAs [27] are derived from introns in protein-coding and noncoding genes transcribed by RNAP II. To address the possibility that some of the TIN transcripts are the sources of these known small RNAs, we compared the human genomic coordinates of TIN sequences to those of 346 snoRNAs [28] and 383 microRNAs [29]. We found that 98 snoRNA or microRNA transcripts (14%) mapped to 86 TIN EST contigs, which may well be the sources of these small RNAs. The 86 TIN EST contigs comprise a very small portion (0.2%) of the TIN transcript dataset. We postulate that the large remaining set could be the source of new snoRNAs and microRNAs as well as of new types of ncRNAs.

### Identification of long, unspliced, partially intronic transcripts

A set of unspliced partially intronic noncoding (PIN) EST contigs was identified. A PIN contig was defined as a contig that overlaps an exon of a RefSeq gene and extends at least 30 bases over both ends of the exon (Figure 1). In total, 12,592 PIN EST contigs (median length 719 nt) were identified. An estimated 90% of PIN transcripts have no or limited protein-coding potential as determined by ESTScan2 analysis. By matching the PIN contig sequences to ESTs from high-quality directionally cloned EST libraries [7], to transcriptionally active regions (TARs) in whole-genome strand specific tiling arrays [30], and to the publicly available unspliced full-length mRNA dataset from GenBank we found that 5,992 PIN contigs (48%) have evidence of being transcribed antisense to the corresponding RefSeq gene. It should be noted that the above EST and tiling array information was not taken as definite evidence of antisense PIN transcription. Sense/antisense PINs were determined experimentally by oligoarray hybridization as described in the following sections, using a pair of separate reverse complementary probes for each PIN in the array, and the strand information was obtained by mapping the actual 60-mer oligonucleotide single-stranded probe to the genomic sequence and recording its strand direction.

### Most RefSeq genes have intronic transcription

Overall, we found that at least 11,679 RefSeq genes, corresponding to 74% of all spliced human genes in the reference dataset, have transcriptionally active introns to which TIN or PIN EST contigs were mapped. If we were to consider TIN or PIN EST singlets, the fraction of RefSeq genes with intronic transcription would increase to 86% of all RefSeq genes.

### TIN and PIN transcripts are potential alternative splicing regulators

We found that the average frequency of exon skipping for genes in the RefSeq reference dataset that show evidence of PIN transcripts is 0.23, and the average frequency of exon skipping for exons immediately 3' to TIN transcripts is 0.22. These frequencies are significantly (*p *< 0.0001) higher than the average frequency of exon skipping (0.14) in the overall set of RefSeq genes (data not shown).

**Figure 2:**
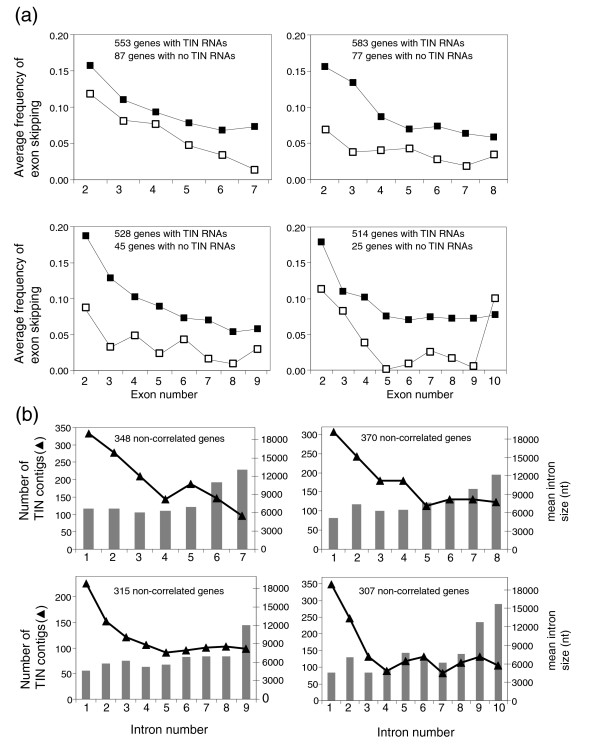
Frequency of exon skipping and abundance of wholly intronic noncoding transcription in RefSeq genes. **(a) **Distribution of exon skipping events along spliced RefSeq genes with 7, 8, 9 or 10 exons. Filled squares indicate the average frequency of skipping per exon for genes with evidence of TIN RNAs mapping to their introns. Open squares indicate the average frequency of skipping per exon for genes with no evidence in GenBank that TIN RNAs map to their introns. A significantly higher (*p *< 0.002) frequency of exon skipping was observed for RefSeq genes with TIN RNA transcription. **(b) **Distribution of TIN transcripts among the introns of RefSeq sequences with 7, 8, 9 or 10 introns selected from GenBank as being outside the 95% confidence level of significance (not correlated) in a Pearson correlation analysis between the abundance of TIN contigs per intron and the intron size (in nt). Bars indicate the average intron size (nt) for this selected set of genes. Triangles indicate the number of TIN contigs per intron for RefSeq genes for the same set.

Next, we examined both the distribution of exon-skipping frequency across the different exons of protein-coding genes (Figure 2a) and the abundance of unspliced TIN EST contigs across the different introns of the same genes (Figure 2b). A higher frequency of exon skipping was detected closer to the 5' ends of protein-coding genes (Figure 2a), and a concomitantly higher abundance of unspliced TIN EST contigs was detected in the first two introns of these genes (Figure 2b). It is known that the average size of first introns is larger than that of other introns when all human genes are considered together. To determine if the higher abundance of TIN contigs in the first introns (Figure 2b) is predominantly due to the longer size of first introns, we separated the genes according to first intron sizes. To that end, we split in two the population of genes with a given number of introns; those where the size of the first intron is similar to the average size of all other introns and those where the first intron is longer than the remaining ones. We found that for the majority of genes with 6 to 12 introns, the average length of the first intron is very similar to the average length of all other introns in the same genes (for example, for genes with 7 introns the fraction is 348/553 = 0.63; Figure 2a,b). For this set of genes, one would expect a random distribution of TIN EST contigs across the different introns if TINs were transcribed by spurious RNAP II transcription. In contrast, we found an uneven distribution of TIN contigs (Figure 2b), which suggests that TIN transcription may frequently be influenced by proximity to the gene promoter and might be regulated and driven by a so far uncharacterized mechanism favoring the first introns. It should be noted that for another fraction of genes with any given number of introns, the first intron is longer than the other introns (for example, for genes with 7 introns the fraction is 168/553 = 0.30), resulting in a significant correlation between frequency of TIN contigs and average intron length (Additional data file 2). The hypothesis is that more information is conveyed in the longer intronic regions of these particular genes (see Discussion).

### Design and overall performance of a gene-oriented intron-exon oligoarray platform

**Figure 3:**
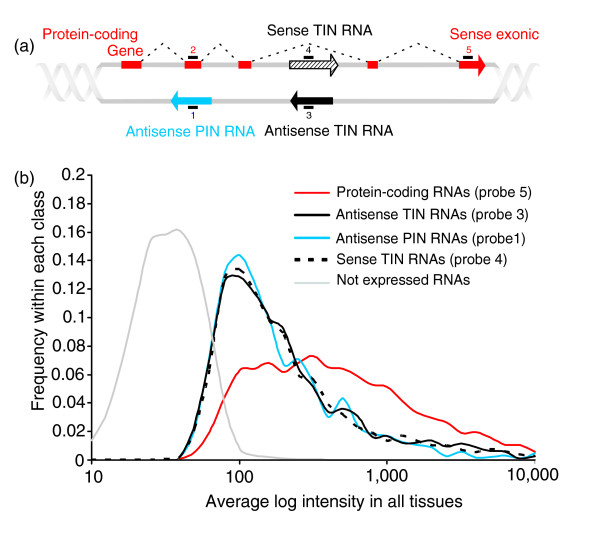
Design and overall performance of the 44 k gene-oriented intron-exon expression oligoarray. **(a) **Schematic view of the 44 k combined intron-exon expression oligoarray 60-mer probe design. Probe 1 is for the antisense PIN transcripts (blue arrow). Probes 3 and 4 are a pair of reverse complementary sequences designed to detect antisense or sense TIN transcripts (black and hashed black arrows, respectively) in a given locus. Sense exonic probes 2 and 5 are for the protein-coding transcripts (red block and red arrow). Note that the latter were not systematically designed for an exon near the TIN message; in most instances a distant, 3' exon of the gene has been probed instead. **(b) **Average signal intensity distribution for antisense TIN (solid black line), sense TIN (dashed line), antisense PIN (blue line), or sense protein-coding exonic (red line) probes. Average intensities from six different hybridization experiments with three different human tissues, namely liver, prostate and kidney, are shown. Only probes with intensities above the average negative controls plus 2 SD were considered. The average intensity distribution for probes below this low-limit detection cutoff is shown in the curve marked as 'Not expressed RNAs' (gray line).

The analyses described so far have indicated the presence of active sites of totally and partially intronic transcription of noncoding messengers (TIN and PIN transcription) within protein-coding genes. Guided by this information, we designed a 44 k intron-exon oligoarray combining randomly selected protein-coding genes along with the corresponding intronic transcripts. This permitted large-scale detection of human intronic expression in a strand-specific, gene-oriented manner. A total of 8,780 probes from the commercially available set of Agilent 60-mer probes (Figure 3a, probe 5) were used, representing different exons in 6,954 unique randomly selected protein-coding genes, along with custom-designed intronic probes for the antisense or sense strand, as shown in Figure 3a. A pair of reverse complementary probes for each of 7,135 TIN transcripts (Figure 3a, probes 3 and 4) was designed, thus independently detecting sense and antisense transcription in a given locus. Probes for 4,439 antisense PIN transcripts (Figure 3a, probe 1) were also designed. A probe representing each PIN-overlapped protein-coding exon was included (Figure 3a, probe 2).

We opted to use the 60-mer Agilent oligoarray technology to construct this custom-designed array because the probe characteristics and the hybridization and washing protocols in this platform have been optimized to attain reproducible results [31]. Therefore, probe design followed Agilent recommendations with respect to GC content and melting temperature (T_m_), as detailed in Materials and methods, to ensure a homogeneous and effective hybridization of fluorescent targets. In fact, the reproducibility of expression in our experiments was fairly high, as evaluated by the correlation coefficients obtained for the two-color raw intensities within each slide and the correlation coefficients of inter-slide comparisons. These correlation coefficients ranged from 0.914 to 0.981 for intra-slide and from 0.915 to 0.949 for inter-slide comparisons.

Probe specificity was ensured by selecting 60-mer sequences with a homopolymeric stretch no longer than 6 bases; in addition, probes should not have 8 or more bases derived from repetitive regions of the genome. The selected probes have a low probability of cross-hybridization, as estimated by a BLAST search against the sequences of all transcribed human messages using the following criteria. All probes have 100% matches to the transcript sequences they represent, which translates into a best-match BLAST bit-score of 119. A bit-score high-end cutoff for the second-best match of each selected probe was set at 42.1, which would correspond to cross-hybridization with a maximum match of 21 bases with no gaps. This high-end cutoff level was determined from the bit-scores of the second-best hits for all the Agilent-designed commercial probes for protein-coding genes included in our platform; it is a conservative cutoff that includes 90% of the Agilent-optimized probes (Additional data file 3). Commercial probes with bit-score cross-hybridization matches higher than 42.1 were included because Agilent have tested each of their probes individually for absence of cross-hybridization [31]. Since we did not test individual probes, we opted to use this conservative high-end cutoff parameter for the intronic probes.

Negative controls in the oligoarray (1,198 Agilent commercial control probes, see Materials and methods) included sequences from adenovirus E1A transcripts, synthetically generated mRNAs, *Arabidopsis *genes and control probes designed not to hybridize to targets because of secondary structure. The hybridization and washing stringency conditions optimized by Agilent ensured that the raw signal intensities for these negative controls (median 34.3) in our experiments were low. For each experiment, the average negative control intensity plus 2 standard deviations (SD) was used as a low-limit cutoff to call the expressed and not-expressed genes.

Figure 3b shows the distribution of average intensities in the microarray experiments for genes called not-expressed (below the low-limit cutoff) and for protein-coding, antisense or sense TIN and antisense PIN expressed transcripts. The distribution is skewed towards higher intensities for protein-coding transcripts and the median intensity is 351. The distribution of intensities is very similar for all types of intronic transcripts, and is skewed towards lower intensities when compared to that of protein-coding genes (Figure 3b). Nevertheless, the median intensities (134 for antisense TIN, 126 for antisense PIN and 135 for sense TIN transcripts) were sufficiently above that of the negative controls to permit a considerable number of expressed intronic transcripts to be detected in all tissues. Discrimination between expressed and not-expressed transcripts may be more critical for intronic messages than for protein-coding ones, and a larger fraction of false-negatives may be present in the intronic data. Our results corroborate previous tiling array measurements in chromosomes 21 and 22 that showed that ncRNAs were generally expressed at lower levels than protein-coding ones [32].

### Partially and totally intronic noncoding transcripts expressed in three human tissues

**Figure 4:**
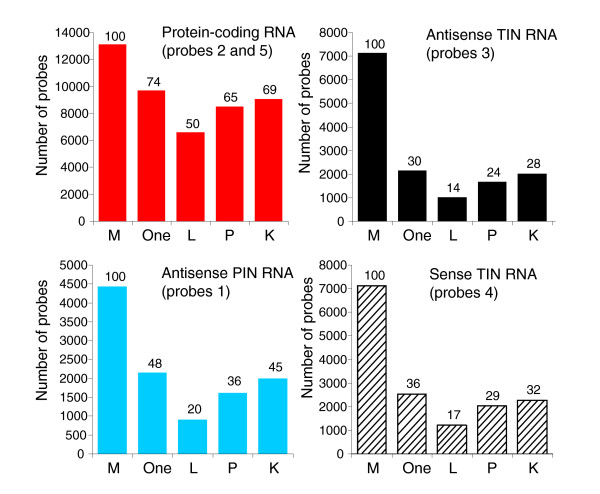
Number of protein-coding, TIN and PIN transcripts expressed in three human tissues. Different types of transcripts are shown in each panel, and are color-coded as in Figure 3: protein-coding exonic (red bars), antisense TIN (black bars), antisense PIN (blue bars) or sense TIN transcripts (hashed black bars). The total number of probes present in the microarray for each type of transcript is shown with bars marked as 'M'. The number of transcripts expressed in at least one of the three tissues tested is shown with bars marked as 'One'. Transcripts exclusively expressed in each of the three tissues are shown with bars marked as 'L' for liver; 'P' for prostate; or 'K' for kidney. The percentage of expressed transcripts relative to the total number of transcripts probed in the array is indicated at the top of each bar.

Gene expression profiles for human prostate, kidney and liver were obtained with the 44 k intron-exon oligoarrays. Arrays were hybridized with amplified Cy3- and Cy5-labeled cRNA obtained by *in vitro *linear amplification of poly(A)-containing RNAs using T7-RNA polymerase. Figure 4 shows the number of protein-coding, TIN and PIN probes with signals greater than the negative control average plus 2 SD in at least one of the three tissues examined, and in each separate tissue. It can be seen that while 74% of protein-coding messages were expressed, only 30% of antisense TIN and 48% of antisense PIN transcripts were expressed in at least one tissue. A similar fraction of sense TIN transcription (36%) was observed, underscoring the natural transcription of sense intronic transcriptional units that has been observed elsewhere [30,33].

It can be seen that 50% to 69% of protein-coding transcripts were expressed in each individual tissue, while 14% t o 32% antisense and sense TIN and 20% to 45% antisense PIN transcripts were detected (Figure 4). This reveals that the abundance of intronic transcripts was lower than that of protein-coding messages, in terms of both the diversity of messages per tissue (Figure 4) and the relative distribution of signal intensities (Figure 3b).

**Figure 5:**
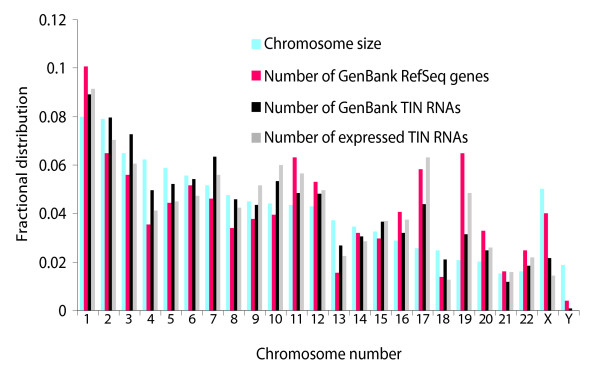
Genomic distribution of intronic RNAs. Relative chromosome sizes (blue bars) and the fractional number of GenBank Refseq genes (red bars) mapped per chromosome are shown. The distribution along the chromosomes of wholly intronic sequence contigs resulting from mapping and assembly of all ESTs in GenBank relative to the RefSeq reference dataset is shown (black bars). The distribution along the chromosomes of intronic RNAs expressed in human liver, as detected by oligoarray hybridizations, is shown as gray ears. The numbers on the y-axis refer to the fractional distribution in each chromosome.

The distribution along human chromosomes of the number of TIN RNA transcriptional units expressed in liver (Figure 5, gray bars) clearly agreed with the distribution computed by informatics analysis based on the entire GenBank EST dataset (Figure 5, black bars). Both distributions generally follow that of the number of RefSeq genes in each chromosome (Figure 5, red bars). There are a few exceptions; for example, chromosomes 10 and 13 seem to contain a higher fraction of expressed TIN RNA transcriptional units than protein-coding RefSeq genes, and chromosomes 19 and X have lower ratios of intronic transcriptional units to protein-coding genes. Interestingly, X chromosome inactivation (XCI) depends on a single noncoding sense-antisense transcript pair, *Xist *and *Tsix*, transcribed from a single locus on chromosome X. At the onset of XCI, *Xist *RNA accumulates on one of the two Xs, coating and silencing the chromosome in *cis*, a phenomenon controlled by a transient heterochromatic state that regulates transcription [34].

**Figure 6:**
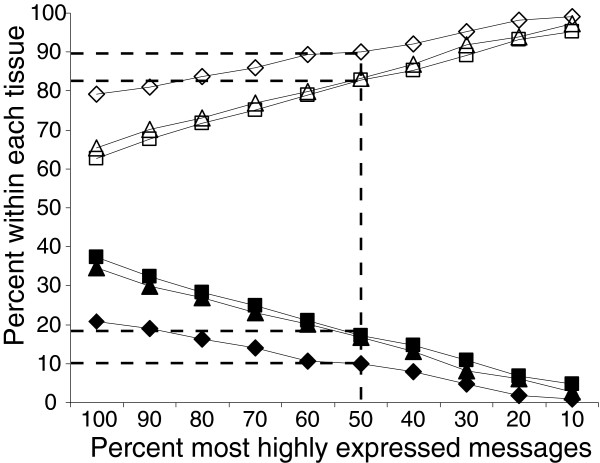
Sense-antisense TIN transcript pairs simultaneously detected at different ranges of signal intensities for each of three different tissues. The percentages of TIN transcript pairs simultaneously transcribed from the same genomic locus in both the sense and antisense orientations (full symbols), and detected at different ranges of signal intensities, are shown for each of three different tissues: liver (diamonds), prostate (triangles) and kidney (squares). The percentages of TIN messages transcribed in each tissue from only one of the two DNA strands (sense or antisense) are shown as open symbols.

Figure 6 shows the distribution of sense and antisense TIN transcripts simultaneously expressed from the same locus as a function of the fraction of transcripts expressed in each of the three tissues. Considering only the top 10% most highly expressed sense and antisense TIN transcripts (the top 10%) in each tissue, only 1% to 5% were detected simultaneously from both strands of the same introns in protein-coding genes. Among the top 50% of intensities, over 83% to 90% of intronic transcription events are specific to one strand. Even when 100% of the expressed transcripts were considered, 63% to 79% were found to be expressed exclusively from one strand. This suggests that most of the sense and antisense messages are independent transcriptional units. It is apparent that the most highly expressed intronic transcripts are strand-specific, which again suggests a regulated cellular process.

### Antisense TIN transcripts are enriched in introns of genes related to regulation of transcription

**Figure 7:**
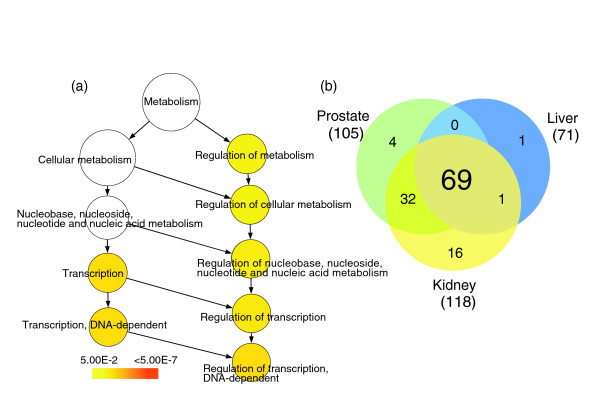
Most highly expressed TIN transcripts map to genes related to regulation of transcription. TIN RNA expression data from three different human tissues (prostate, liver and kidney) were used to select the protein-coding genes to which the top 40% most highly expressed TIN transcripts map. The BiNGO program was used to identify significantly (*p *≤ 0.05) enriched GO terms within the set of selected protein-coding genes. **(a) **GO-enriched categories for prostate are shown in color, which is related to the *p *value as indicated by the color-code bar. The exact *p *values for all significantly enriched GO categories are shown in Additional data file 4. GO category 'Regulation of transcription, DNA-dependent' (GO:006355) is the most significantly enriched (*p *= 0.002). Similar results were obtained for liver and kidney (see Additional data file 4). **(b) **Venn diagram for the 123 unique protein-coding genes belonging to GO:006355 category 'Regulation of transcription, DNA-dependent'. The number of genes in each tissue for which intronic transcription was detected is shown in parenthesis; the numbers of coincident and dissimilar genes among kidney, prostate and liver are shown in the circles.

We selected the top 40% most highly expressed antisense TIN transcripts in each tissue and identified the protein-coding genes to which these transcripts map. The GO annotation of these protein-coding genes was compared with the BiNGO tool [35] to the entire list of protein-coding genes in the array that showed evidence of antisense TIN transcription. The GO category 'Regulation of transcription, DNA-dependent' (GO: 006355) was found to be significantly enriched in prostate (*p *= 0.002), kidney (*p *= 0.002) and liver (*p *= 0.022). A typical GO enrichment analysis is shown for prostate in Figure 7a; similar results for kidney and liver are shown in Additional data file 4. The exact *p *values for all significantly enriched GO categories can be found in Additional data file 4.

Among the top 40% most highly expressed antisense TIN transcripts mapping to 678 protein-coding genes in the prostate, 105 (16%) belong to 'Regulation of transcription, DNA-dependent' (Figure 7b). Analogous results were obtained for liver and kidney, where 71 out of 409 (17%) and 118 out of 812 (15%) of the genes, respectively, belong to 'Regulation of transcription, DNA-dependent'. A total of 123 unique genes related to 'Regulation of transcription' were found in common among the 40% most highly expressed antisense TIN transcripts in prostate, kidney or liver. Most of these (69 genes, 56%) were expressed in all three tissues (Figure 7b), while some were shared between two tissues and a few were only expressed in one. The 'Regulation of transcription' GO category includes genes encoding various DNA-binding proteins such as transcription factors, zinc fingers and nuclear receptors. The entire list of genes identified in Figure 7b can be found in Additional data file 5. Similar analyses with the top 40% highly expressed sense TIN and antisense PIN transcripts did not identify any enriched GO category.

A similar analysis using the top 40% most highly expressed protein-coding genes showed an entirely different set of significantly (*p *< 0.05) enriched GO categories; between 10 and 15 significantly enriched categories were detected in each tissue, and none was related to 'Regulation of transcription' (Additional data file 6). The most significantly enriched GO categories in all three tissues include genes involved in RNA and protein biosynthesis, ribosome biosynthesis, mRNA processing and initiation of translation.

### Many TIN and PIN RNAs are insensitive to RNAP II inhibition or are even up-regulated by α-amanitin

**Figure 8:**
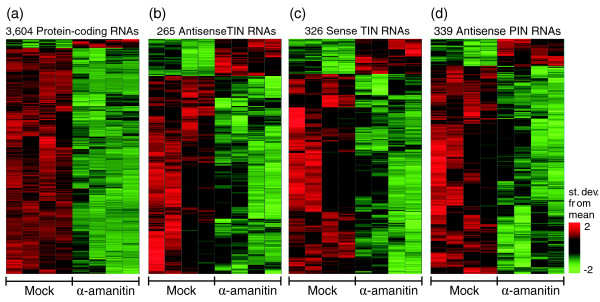
Effect of RNAP II inhibitor α-amanitin on the abundance of protein-coding, antisense TIN, sense TIN and antisense PIN RNAs. Lines on each panel represent various transcripts for which the expression levels differed significantly between α-amanitin-treated prostate cells and untreated control cells. Each sample replica is shown in one column. Transcripts were selected by a SAM two-class test (FDR <0.2% to 2%) combined with a signal-to-noise test (*p *≤ 0.05). For each line, expression intensities were normalized between the two conditions and colored as a function of the number of standard deviations from the mean value; (a) 3,604 significantly affected protein-coding transcripts; (b) 265 significantly affected antisense TIN transcripts; (c) 326 significantly affected sense TIN transcripts; (d) 339 significantly affected antisense PIN transcripts.

We treated human prostate cancer-derived LNCaP cells with the RNAP II inhibitor α-amanitin for 24 hours, and used the 44 k oligoarray to assess its effect on the expression of protein-coding and noncoding intronic RNA. Differentially expressed transcripts (Figure 8) were identified by combining two statistical approaches, the significance analysis of microarray (SAM) method with a false discovery rate (FDR) <2% [36] and a signal-to-noise ratio (SNR) analysis with bootstrap permutation (*p *< 0.05) [37]. About 39% (3,604) of the expressed protein-coding messages were significantly affected by RNAP II inhibition, while the remaining presumably more stable mRNAs were not. As expected, most (96%) of the affected protein-coding messages were down-regulated, but 4% were up-regulated. We found that 129 protein-coding RNAs were up-regulated at least two-fold. Kravchenko *et al*. [23] found that a similar number of protein-coding RNAs (70 transcripts) were up-regulated two-fold or more by α-amanitin in HeLa cells in experiments with Affymetrix oligoarrays representing approximately 20,000 protein-coding transcripts.

Markedly fewer of the expressed TIN antisense (12%) and sense (14%) transcripts were affected by α-amanitin. Similar fractions of antisense (16%, 42/265) and sense (15%, 49/326) TIN transcripts were up-regulated in α-amanitin treated cells (Figure 8). PIN antisense transcript levels exhibited an expression pattern rather different from that of protein-coding transcripts when RNAP II was inhibited: only 15% were affected, of which 12% (39/339) were up-regulated. Interestingly, 3 to 4 times as many TIN and PIN RNAs as protein-coding messages (4%) were up-regulated by α-amanitin (Figure 8).

**Figure 9:**
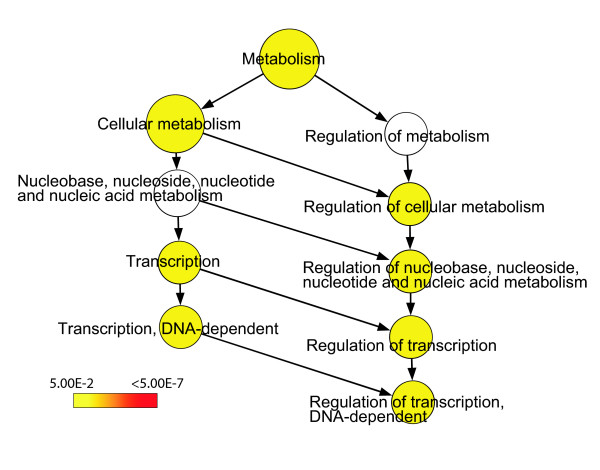
Genes with increased intronic transcription in the presence of the RNAP II inhibitor α-amanitin are enriched in the 'Regulation of transcription' GO category. Gene ontology analysis was performed on protein-coding genes that were shown in the experiment illustrated in Figure 8 to have up-regulated expression of antisense PIN transcripts and sense and antisense TIN transcripts upon exposure to α-amanitin. Significantly (*p *≤ 0.05) enriched GO terms are shown in color, which is related to the *p *value as indicated by the color-code bar. The exact *p *values for all significantly enriched GO categories are shown in Additional data file 7.

Intriguingly, the intronic messages (both TIN and PIN transcripts) with significantly increased abundance in cells with blocked RNAP II transcription were transcribed from the introns of protein-coding genes that are again enriched in the 'Regulation of transcription' GO category (*p *= 0.02; Figure 9). A complete list of the noncoding intronic and protein-coding transcripts that were up-regulated upon exposure to α-amanitin and the exact *p *values for all significantly enriched GO categories are shown in Additional data file 7.

We consider that the stringent criteria used, combining two statistical methods to identify the differentially expressed transcripts, may be conservative. Therefore, the proportion of intronic messages that are up-regulated following α-amanitin treatment may be even greater than those reported here. In any case, the number of intronic ncRNAs insensitive to inhibition, or up-regulated upon α-amanitin treatment, is likely to be in the thousands when extrapolated to all the intronic transcripts found in human cells. Considering only the 55,139 wholly intronic EST clusters, over a thousand are predicted to be up-regulated if at least 13% are affected by 24 hours of RNAP II inhibition.

### Tissue signatures of TIN and PIN expression

**Figure 10:**
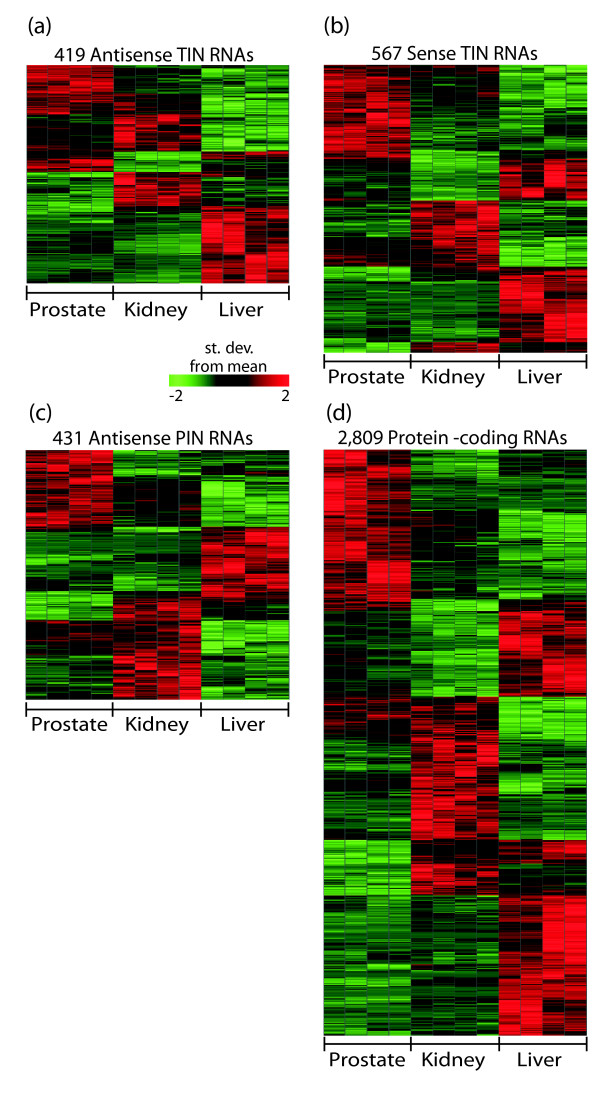
Expression signature of intronic and protein-coding transcripts in human liver, prostate and kidney. Transcripts with significantly different levels among prostate, kidney and liver samples were selected by a SAM multi-class test (FDR <0.002) combined with an ANOVA test (*p *≤ 0.001) and hierarchically clustered as described in the Materials and methods. In each panel the selected transcripts are shown in the lines and sample replicas in the columns. For each line, expression intensities among the three tissues were normalized within each type of probe and colored as a function of the number of standard deviations from the mean value. **(a) **Tissue expression signature of 419 antisense TIN transcripts. **(b) **Tissue expression signature of 567 sense TIN transcripts. **(c) **Tissue expression signature of 431 antisense PIN transcripts. **(d) **Tissue expression signature of 2,809 protein-coding transcripts.

Tissue-specific signatures of intronic expression were determined for prostate tumor, normal kidney and normal liver. A total of 419 antisense TIN (Figure 10a), 567 sense TIN (Figure 10b) and 431 antisense PIN (Figure 10c) transcripts were identified, using a combination of two statistical approaches (see Materials and methods for details). A complete list of the intronic transcripts identified in tissue signatures, and the corresponding spliced protein-coding genes mapping to the same genomic loci, is provided in Additional data files 8-10. These tissue signatures comprise hundreds of different transcripts (Figure 10a-c) mapping to introns of genes with diverse functions, and no particular GO category enrichment could be detected.

A tissue signature containing 2,809 protein-coding transcripts was also identified (Figure 10d). Analysis of GO enrichment (not shown) revealed that in liver the protein-coding tissue signature is enriched in GO categories related to urea cycle (GO: 006594), cysteine metabolism (GO: 006534), cholesterol biosynthesis (GO: 008203) and prostaglandin metabolism (GO: 006693), while in kidney it is enriched in the GO categories related to sodium and potassium ion transport (GO: 006834 and GO: 006813, respectively). In the prostate, no relevant GO categories were enriched, but prostate-specific genes such as *KLK3 *and *TMEPAI *were found.

**Figure 11:**
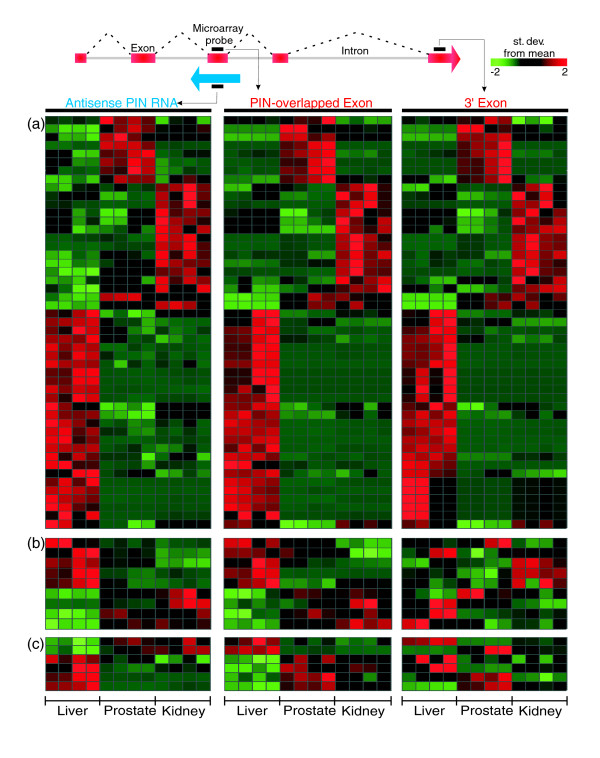
Expression signatures of antisense PIN RNAs and corresponding PIN RNA-overlapped exon pairs relative to their 3' protein-coding exons. A subset of 64 pairs of antisense PIN RNAs and corresponding PIN RNA-overlapped exons were identified among the tissue signatures shown in Figure 10 as having correlated patterns of expression: **(a) **49 pairs were identified in which the 3' exon of the protein-coding transcript (right panel) follows a similar expression pattern to that of the PIN RNA/PIN RNA-overlapped exon pair (left and central panels); **(b) **9 pairs were identified in which the 3' exon of the protein-coding transcript (right panel) does not follow the pattern of tissue expression of the PIN RNA and the corresponding PIN RNA-overlapped exon (left and central panels); **(c) **6 pairs in which the PIN RNA (left panel) has an expression pattern inverted in relation to that of the PIN RNA-overlapped exon (central panel). Each line represents a genomic locus covered by three different types of probes (antisense PIN RNA, PIN RNA-overlapped protein-coding exon and 3' protein-coding exon). For each line, expression intensities among the three tissues were normalized within each type of probe and colored as a function of the number of standard deviations from the mean value.

We searched for co-regulated intronic and protein-coding pairs of messages that were simultaneously expressed from the same genomic locus in the same tissue, in order to identify noncoding RNAs potentially involved in modulating gene expression in a *cis*-acting manner. For this purpose, we initially cross-referenced the tissue signature of antisense PIN RNAs (Figure 10c) with the protein-coding signature (Figure 10d) to determine whether both signatures contained PIN-overlapped exons of the protein-coding gene transcribed from the opposite strand in the same genomic locus (Figure 3, probe 2). Considering all three tissues, we found 64 gene loci in which antisense PIN RNAs and PIN RNA-overlapped protein-coding exon pairs were simultaneously detected in both tissue signatures (Figure 11). The tissue expression patterns of PIN RNA and PIN RNA-overlapped exon pairs were similar in a subset of 49 loci (Additional data file 11; Figure 11a, left and central panels). Interestingly, the 3' exon of the protein-coding transcript in this subset (Figure 11a, right panel) follows the same pattern. This is the predominant pattern in the tissue signature. Conceivably, the similar relative levels of antisense PIN RNA and protein-coding exons indicate that the intronic RNA has a functional role in modulating the transcription or transcript stability of the corresponding protein-coding gene. Alternatively, the levels of antisense PIN RNA and protein-coding message in each tissue may be similar because a common factor simultaneously modulates the transcription of both types of message from the same locus.

In a smaller subset of nine loci, the 3' exon of the protein-coding transcript (Figure 11b, right panel) does not follow the pattern of tissue expression of the PIN RNA and the corresponding PIN-overlapped exon of the protein-coding gene (Additional data file 11; Figure 11b, left and central panels). In addition, the PIN RNA (Additional data file 11; Figure 11c, left panel) in six loci has an inverted expression pattern relative to that of the PIN RNA-overlapped exon (Figure 11c, central panel). In some tissues, there is an inverted pattern in the relative levels of PIN-overlapped exon and the 3' exon of the protein-coding gene for these two sets (Figure 11b,c, central and right panels), suggesting that the protein-coding message is alternatively spliced in a tissue-dependent manner. The similar levels of PIN RNAs and PIN-overlapped exons in Figure 11b (central and right panels) suggest that, in these cases, the PIN RNA may be involved in exon retention of the protein-coding gene, whereas the inverted pattern observed in Figure 11c (central and right panels) suggests that the PIN RNA may favor skipping of the overlapped exon. The effect of intronic RNAs on splicing has been documented in a recent report, where overexpression of a naturally occurring antisense PIN RNA (*Saf *transcript) mapping to the first intron of *Fas *caused the retention of an alternative *Fas *exon that was complementary to the antisense PIN transcript [17].

**Figure 12:**
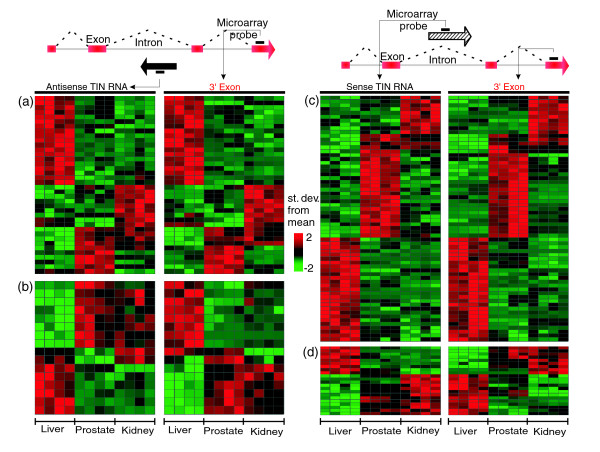
Expression signatures of wholly intronic RNAs relative to their 3' protein-coding exons. Cross-referencing of the tissue signatures shown in Figure 10 identified subsets of TIN RNAs that have correlated patterns of expression relative to the 3' protein-coding exon signature from the corresponding genomic loci: **(a) **38 pairs were identified in which the 3' exon of the protein-coding transcript (right panel) follows a similar expression pattern to that of the antisense TIN RNA (left panel); **(b) **16 pairs were identified in which the 3' exon of the protein-coding transcript (right panel) follows a pattern of tissue expression inverted in relation to that of the antisense TIN RNA (left panel); **(c) **64 pairs were identified in which the 3' exon of the protein-coding transcript (right panel) follows a similar expression pattern as that of the sense TIN RNA (left panel); **(d) **22 pairs were identified where the 3' exon of the protein-coding transcript (right panel) follows a pattern of tissue expression inverted in relation to that of the sense TIN RNA (left panel). For each line in each panel, expression intensities among the three tissues were normalized within each type of probe and colored as a function of the number of standard deviations from the mean value.

An analogous cross-reference of tissue signatures from intronic and protein-coding messages (Figure 10d) was performed using the antisense and sense TIN RNA tissue signatures (Figures 10a,b). Among the three tissues, we compiled 140 gene loci in which pairs of antisense or sense TIN RNAs and the 3' protein-coding exon were simultaneously detected in the tissue signatures (Figure 12). A similar tissue expression pattern of antisense TIN RNA and the 3' protein-coding exon pair was detected in a subset of 38 loci (Additional data file 12; Figure 12a). For 16 pairs of antisense TIN/3' exon an inverted expression pattern was observed (Figure 12b). Similar direct or inverted expression patterns were found for 64 (direct) or 22 (inverted) pairs when sense TIN and 3' protein-coding exons from the same locus were cross-referenced (Figure 12c,d, respectively). The slightly higher proportion (64/86 = 0.74) of directly correlated sense TIN/3' exon pairs compared to antisense TIN/3' exon pairs (38/54 = 0.70) may have resulted from internal priming of intronic segments of premature mRNAs containing stretches of poly(A) during cRNA amplification when the target was being prepared. As for co-regulated PIN RNAs, the correlated expression of TIN RNAs and the 3' exons of protein-coding transcripts may suggest that these noncoding RNAs have a role in modulating the transcription rate or the stability of the corresponding protein-coding RNA.

## Discussion

### Long intronic unspliced transcripts in humans

In this work we have evaluated the contribution of introns in the human genome to the production of noncoding RNAs by gathering data on expressed intronic sequences from public databases, and in parallel by measuring expression with combined intron-exon oligoarrays. We focused on the unspliced messages that map totally (TIN) or partially (PIN) to intronic regions and found that most of the genes defined by RefSeq sequences (74%) undergo intronic transcription. This fraction is likely to prove even greater since intronic expression has not yet been assessed in different developmental stages and physiological conditions. While some of the unspliced intronic ESTs (mapping to the sense strand) may represent hitherto-overlooked exons of alternatively spliced forms of known genes, a significant number of the sense and antisense transcripts in this dataset is likely to derive from novel independent transcriptional units. This is supported by the low protein-coding potential and long length of the TIN and PIN EST contig sequences (medians of 573 nt and 719 nt, respectively), well above the typical lengths of exons of protein-coding genes (median 141 nt).

The median length of the 55,000 TIN RNAs identified in all chromosomes in our analysis is in line with the lengths observed in previous reports by RACE analysis of non-annotated transcripts from 10 human chromosomes (average length 680 nt, range 173 to 4,650 [38]). Almost none of the TIN EST sequences (0.2%) matched known snoRNAs or microRNAs. Nevertheless, it remains possible that some long TIN messages are precursors of yet-undiscovered small RNAs.

We found no correlation between intron size and the abundance of mapped TIN unspliced EST contigs for most of the genes (approximately 60%) that showed evidence of intronic transcription, suggesting that most intronic transcription does not occur by chance. In addition, the consistent correlation between approximately 30% of TIN contigs and intron length might support the 'genomic design' hypothesis [39,40], in the sense that transcription of the longer introns in tissue and development-specific genes could carry regulatory information [39,40]. In effect, we see a more abundant expression of intronic antisense messages in genes with regulatory functions (see discussion below).

We have shown that long TIN RNAs were correlated to the degree of malignancy in prostate cancer [19]. To investigate if there is a preferential contribution of ESTs from tumor libraries to the set of TINs identified in this work, we compiled the information regarding normal or neoplastic tissue origin that is documented in the Cancer Genome Anatomy Project (CGAP) database [41], and assigned it to the set of five million ESTs analyzed in this work. We found that 43% and 57% of the 5 million ESTs are derived from tumor or normal libraries, respectively. Interestingly, we found that the same distribution (43% and 57%) was present in the set of 190,583 ESTs included in the TIN contig dataset. Therefore, there is no biased contribution from tumor EST libraries to the TIN dataset. Moreover, we found that 49% of the 55,139 TIN contigs contained at least one EST from a tumor library, suggesting that TIN transcription is equally present in normal and tumor tissues. These results corroborate the notion that TIN transcription is not an exclusive feature of neoplastic tissues, but rather part of the normal transcriptional output of the cells that may be partially dysfunctional in cancer disease.

### Intronic transcripts may stabilize protein-coding transcripts or regulate their alternative splicing

Most of the PIN and TIN RNAs selected in the tissue-specific signatures have the same tissue expression patterns as the corresponding protein-coding genes. This might indicate that transcription of some PIN and TIN RNAs is linked to a *cis*-acting stabilization of the corresponding protein-coding transcript [42-44]. Intronic transcripts may also act in *trans*, for example, by controlling regional chromatin architecture as demonstrated for some specific long ncRNAs [34,45,46]. Overexpression of complete introns in the *CFTR *gene affects the expression of a large number of protein-coding messages in *trans*, many of them related to *CFTR *function [47].

A few PIN RNAs selected in the tissue-specific signatures showed tissue expression patterns that correlated with the corresponding protein-coding exon overlapped by the PIN transcript. However, the expression pattern of an exon closer to the 3' end of the same protein-coding gene was not correlated, suggesting that alternatively spliced isoforms of the protein-coding transcripts were tissue-specific. Splicing is known to be modulated by the binding of intronic splicing enhancer (ISE) and silencer (ISS) elements to regulatory factors, favoring or blocking spliceosome formation [48]. Some PIN RNAs might regulate the skipping or retention of exons by interacting either with splice signals or with ISE and ISS elements in pre-mRNAs. In fact, there are examples of control of exon skipping by artificially introduced oligonucleotides in human cells [49], and some promising therapeutic strategies rely on antisense oligonucleotides that modulate exon-skipping [50]. As for exon retention, a recent report has identified a long antisense noncoding transcript named *Saf*, which maps as a partially intronic transcript to the first intron of *Fas*, a gene encoding an apoptotic protein [17]. Overexpression of *Saf *in Jurkat cells induced the expression of different alternatively spliced *Fas *isoforms, in which an alternative exon overlapped by *Saf *was retained and non-adjacent 3' exons were skipped, indicating that *cis*-acting antisense intronic RNAs have a regulatory function [17].

Our present microarray analysis is conservative, in that we only selected transcripts that had correlated patterns of intronic and protein-coding messages and were also simultaneously present in tissue-signatures. A more direct experimental approach, for example, over-expressing or suppressing specific PIN transcripts and measuring their effect on the splicing pattern of the overlapped exons, might reveal novel candidates for antisense RNA regulators of exon usage that possibly contribute to a ubiquitous and under-appreciated mechanism of alternative splicing regulation.

Alternative splicing affects more than 70% of human protein-coding genes [51], in which exon skipping is the most frequent event [52]. We found *in silico *evidence that *cis*-acting intronic transcription influences alternative splicing, that is, a higher incidence of noncoding transcription in the first introns along with higher skipping frequency of the first exons in the protein-coding genes. In addition, the frequency of skipping for exons close to or overlapped by intronic transcripts was significantly higher than the average frequency of exon skipping in the overall set of human genes. In fact, exon skipping can be artificially induced by introducing antisense oligonucleotides that map to intron/exon junctions [49,50] or to wholly intronic regions [48].

The higher incidence of transcription in the first intron, closer to the gene promoter, might have other functional implications, such as the impairment of transcription by transcriptional interference [53,54], regulation of gene promoter usage [55], or regulation of the initiation of RNAP II transcription [15]. In the latter case, ncRNAs are known to function as co-activators; protein-binding ncRNAs are expected to provide a broad and diverse way of controlling mRNA transcription [15,56]. We speculate that a high fraction of the intronic transcripts, especially the sense TIN RNAs, may act in *trans*, being parts of multi-component RNA-protein complexes that regulate gene expression. There are thousands of potential RNA regulators, which may effectively amplify the complexity of a human genome with a limited number of protein-coding genes [11,57] through RNA-RNA, RNA-DNA, or RNA-protein interactions.

### Biogenesis of TIN and PIN transcripts

We evaluated the contribution of RNAP II to the biosynthesis of intronic ncRNAs in human cells by blocking its activity with α-amanitin and measuring the levels of protein-coding and noncoding intronic messages. Remarkably, a considerable fraction (12% to 16%) of the wholly intronic or partially intronic antisense transcripts was up-regulated, a fraction 3- to 4-fold higher than that observed for protein-coding messages (4%). In addition, fewer intronic (12% to 15%) than protein-coding (39%) transcripts were sensitive to RNAP II inhibition. Importantly, the sense TIN RNAs responded quite similarly to antisense TIN RNAs with respect to RNAP II inhibition, suggesting that these ncRNAs share similar properties that are different from protein-coding messages. The refractory behavior of intronic transcript expression after α-amanitin treatment, and the apparent up-regulation of many intronic transcripts, suggests that a different transcriptional system may be involved in the biosynthesis of these long wholly intronic ncRNAs. A reasonable candidate is spRNAP IV, which is activated by α-amanitin [23], though the mechanism involved remains elusive. Further experimentation is warranted to verify this hypothesis.

### Advantages of a gene-oriented combined intron/exon expression array platform

Experimental analysis using genome tiling arrays has permitted unbiased probing of transcribed regions in the human genome [32,33,38,58,59]. Probing chromosomes 21 and 22 revealed 5.3 kb of novel transcribed sequences within or overlapping the intronic regions of well-characterized genes, of which 2.7 kb (51%) are antisense to the protein-coding genes [32]. Tiling arrays of the whole human genome have extended these analyses, detecting messages in liver that map to 1,529 and 1,566 novel intronic transcriptionally active regions (TARs) arising, respectively, from the antisense or the sense strands of the corresponding gene [30]. Genome tiling arrays for 10 human chromosomes revealed interlaced networks of both poly A^+ ^and poly A^- ^annotated transcripts and unannotated transcripts of unknown function [38]. It has become apparent that introns as well as intergenic regions constitute major sources of non-protein-coding RNAs [44], and tiling arrays promise to help unravel the complex cellular program of intronic transcription.

Different physiological and pathological conditions are yet to be probed by tiling arrays, and the amount and complexity of the information generated by high-density whole human genome tiling arrays may make the experiments difficult to perform. In this context, we believe that a gene-oriented combined intron-exon expression array that samples the intronic noncoding regions of the genome from which there is previous evidence of transcription, along with the corresponding protein-coding regions, will help to identify the particular gene families, biological processes or functional gene categories of greatest relevance to any physiological condition under study. In the present case, we have opted to probe in a combined intron-exon oligoarray approximately 15% (7,135 TINs) of the 55,139 wholly intronic genomic regions with evidence of transcription. With such a platform we were able to interrogate the intronic expression of three different tissues, and we found 1,915 sense and antisense TIN transcripts expressed in liver, 3,288 in prostate and 4,012 in kidney (Figure 4). A total of 4,296 unique intronic regions (60% of all probed TIN loci) were actively transcribed in at least one tissue, as determined by our combined intron-exon expression oligoarray. Thus, it is apparent that most of the 55,139 intronic regions with evidence of transcription from EST and mRNA data can be independently confirmed by direct hybridization, pointing to the best candidate set of intronic genomic regions to be studied in more detail. High-density custom tiling arrays of selected chromosome regions containing genes that are identified as preferentially transcribed in a given tissue should permit further detailed studies of intronic expression patterns. The information gathered from such complementary approaches should help accelerate the acquisition of information about the emerging diverse roles of intronic messages.

### Tissue-specific intronic expression and enrichment of genes related to regulation of transcription

Tissue-specific expression signatures provide strong evidence that intronic transcripts are physiologically relevant. Expression signatures of microRNAs have been reported to classify human cancers [22], adding to the evidence that different ncRNAs are tissue-specific and functionally important.

The present finding that the most abundant wholly intronic antisense RNAs are transcribed from introns of genes related to the regulation of transcription provides a clue to their functional relevance. A high degree of conservation is expected in those intronic genomic regions that are under strong selective constraints. In fact, conserved genomic regions have been identified by several different approaches in the introns of genes involved in transcriptional regulation [60-64]: identification of non-transcribed ultraconserved sequences [60], multispecies conserved sequences [61], sequences conserved in vertebrates but highly divergent among chimpanzees and humans [62], short blocks of multiple-copy sequences (pyknons) [63], or conserved regions without transposon insertions [64]. Our results add to these findings by showing that conserved intronic DNA segments of genes involved in transcriptional regulation are the sources of one of the most abundant intronic RNAs in three different human tissues. The possibility that regulatory genes are controlled by ncRNAs transcribed at the same loci is appealing. It would represent an additional mechanism for regulating the regulators, in a rather sophisticated system for fine-tuning eukaryotic gene expression.

## Conclusion

Our approach has used an oligoarray-based gene-oriented combined intron-exon expression platform as a practical and effective compromise between a biased exon array that only probes the protein-coding messages, and the whole human genome tiling arrays. This approach has identified potentially functional intronic RNAs that are most abundantly transcribed from introns of genes involved in transcriptional regulation. Further comparative analysis of intronic transcription under a different number of physiological and pathological conditions should advance current knowledge about the diverse biological roles of these noncoding RNAs in the control of gene expression.

## Materials and methods

### Cross-referencing of genomic coordinates of transcripts from different sequence datasets

The analyzed sequence dataset comprises all human RefSeq, mRNA and EST sequences, of which the genome coordinates were downloaded from the Genome Browser web page [65] (hg17; NCBI Build 35, March 2005). First, sequences with poor alignment quality (coverage <0.70 and identity <0.90) or mapped to more than one genomic region were removed. Second, we discarded sequences with complicated rearrangement patterns, such as T-cell receptor and immunoglobulin genes. ESTs and mRNAs that aligned to exons of two or more non-overlapping RefSeqs from the same genomic strand were filtered out as suspected chimeras. Sequencing errors in transcripts aligned to the genome sequence led to gaps that are interpreted as introns by our parser. To avoid these falsely identified introns, we joined adjacent exons whenever an intron of less than 30 bases was detected.

A bioinformatics tool was developed to handle the over five million human ESTs efficiently. Essentially, this tool consists of a package of scripts written in Perl that uses files of genome mapping coordinates directly obtained from the UCSC genome browser. The use of coordinates avoids the computationally intensive and parameter-dependent problems of alignment-based programs. EST sequences with overlapping exons were merged into EST clusters using the genomic mapping coordinates. RefSeq and mRNA sequences were processed separately and split into four sets according to the genomic strand to which they mapped, and further sub-divided into spliced and unspliced groups of messages. Sequences from the same strand in each subgroup were merged into a transcriptional unit when their exons overlapped at the same genomic locus. The mRNA dataset was aligned against the RefSeq dataset to identify additional splice variants, intronic and antisense transcripts represented in the mRNA collection, as detailed in Table 1. A complete list of sense/antisense transcript pairs identified here is given in Additional data file 1.

From the combined data described above, a reference dataset was defined comprising the set of 15,783 spliced non-redundant RefSeq transcriptional units plus the evidence of additional splice variants obtained for each transcriptional unit from all mRNA sequences mapping to the same locus. As a control to our filter and clustering procedures this reference dataset was cross-referenced to the lists of previously known sense/antisense pairs [8,9]. First, we eliminated from the published lists of pairs those that were composed of sequences that had been eliminated from the UCSC hg17 database and, therefore, were not by definition in the dataset analyzed here (181 pairs from [8], and 15 from [9]). Second, we eliminated from the published lists those pairs for which there was no evidence of sense/antisense overlap from RefSeq or mRNA, only from ESTs, since this was the criterion used in the present analysis to establish our reference dataset (1,002 pairs from [8] and 822 from [9]). Next, we found 45 sequences from [8] and 159 from [9] that matched clusters in our dataset that contained only mRNAs, not RefSeq sequences. The remaining 1,432 pairs from [8] and 1,740 from [9] comprise the pairs that were expected to be found in our RefSeq reference dataset. Of these, a total of 1,429 (99.8%) from [8] and 1,734 (99.7%) from [9] were covered by our dataset; only 3 pairs from [8] and 6 from [9] were filtered out from our dataset because of various low quality criteria that we had implemented (see filters above).

Subsequently, this RefSeq reference dataset was compared to the total set of EST clusters in order to define those that were exonic or wholly intronic to genes in the reference dataset. The genome mapping coordinates of each of the 55,139 unspliced EST contigs identified as wholly intronic to RefSeq genes (TIN RNAs, Table 2) are listed in Additional data file 13, and the file is formatted in a way that each entry can be uploaded as a track in the UCSC genome browser tool hg17 assembly version of May 2004 and viewed. For wholly intronic RNAs, we recorded the relative position of the RefSeq intron to which the RNA mapped with respect to the total number of introns of the respective RefSeq reference gene.

Partially intronic EST contigs were identified in a later step, by searching for evidence of two or more overlapping EST sequences that mapped to an exon and covered the intronic regions flanking the exon on each side by more than 30 contiguous bases (unspliced extension of the exon). The genome mapping coordinates of each of the 12,592 EST contigs identified as partially intronic to RefSeq genes (PIN RNAs) are listed in Additional data file 14, and the file is formatted in a way that each entry can be uploaded as a track in the UCSC genome browser tool hg17 assembly version of May 2004 and viewed.

Only the genomic mapping coordinates of TIN and PIN contigs were recorded, not the genomic strand orientation; direct experimental determination of strandedness of transcription was obtained by oligoarray hybridization, using a pair of separate reverse complementary probes for each TIN or PIN in the array as described in the following sections.

### Exon skipping frequencies

For each exon of a gene from the RefSeq reference dataset, we counted the number of times that it mapped to an exon (# in exon) or an intron (# in intron) in all the mRNA sequences from the same subgroup (mRNAs and RefSeqs from the same locus and on the same genomic strand). Exon skipping (ES) frequency was given by:

ES = 1 - [# in intron/(# in intron + # in exon)]

### Design of the 44 k intron-exon oligoarray

Oligonucleotide probes were designed for the sense and antisense strands of each of 7,135 totally (TIN) and 4,439 partially (PIN) intronic noncoding RNAs picked randomly from the list of unspliced EST contigs with most abundant ESTs representing each type of intronic transcript. First, for each PIN or TIN RNA, we selected all 60-mer sequences that satisfied a series of conditions [31] as follows: probes should not have 8 or more bases derived from repetitive regions of the genome or homopolymeric stretches of 7 or more bases (low complexity); and they should have a GC content of 35% to 55% and a Tm of 68-76°C. To reduce cross-hybridization, each 60-mer sequence was searched by BLAST against a specific database comprising all human genomic regions for which the mRNA or EST data give any evidence of transcription. Those 60-mer sequences for which the second best hits against this database had bit-scores equal to or lower than 42.1 were carried forward to the next step. They were mapped back to their respective targets and one probe was selected closer to the 3' end of each target in the antisense direction, relative to the protein-coding genes. For each target, a second probe was selected for the opposite strand by taking the reverse complementary sequence of the selected 60-mer, so that a pair of sense/antisense probes is present for each TIN and PIN candidate region in the array. For PIN RNAs, the probe on the opposite strand corresponds to the exon of the gene where the partially intronic message overlaps. To measure the transcriptional level of the protein-coding genes to which PIN and TIN RNAs mapped, we included in our array 14,074 elements corresponding to exons of 7,464 unique Agilent-designed probes contained in the Whole Human Genome Oligo Microarray set (matched by their Gene_Name annotations to the RefSeq reference dataset genes to which the PIN and TIN RNAs mapped), together with the set of 2,256 positive and negative control Agilent commercial probes (IS-44290-1-V1_eQC-V1) designed for the Agilent human expression oligoarrays. Our custom-designed 44 k intron-exon oligoarrays were printed by Agilent Technologies. A list of all probes is available at GEO under accession number GPL4051, and also as Additional data file 15. The genome mapping coordinates of each of the intronic and exonic oligoarray probes are listed in Additional data file 16, and the file is formatted in a way that each entry can be uploaded as a track in the UCSC genome browser tool hg17 assembly version of May 2004 and viewed.

### Human tissue samples

Total RNA was purified from two pools of normal human liver, each with samples from 5 individuals, and from normal kidney tissues obtained from 17 individuals. Four pools of normal kidney were prepared (three with four samples and one with five samples). In addition, two prostate tumor samples were used. All samples were obtained from patients who signed informed consent, and approval was received from the ethics committees of the hospitals. Total RNA was purified using Trizol (Invitrogen, Carlsbad, CA, USA) according to the manufacturer's instructions, followed by treatment with DNase I following the 'on-column DNase digestion' protocol of the Qiagen RNeasy kit (Qiagen, Valencia, CA, USA) to remove potential genomic DNA contamination. All RNA samples were checked for purity using a ND-1000 spectrophotometer (NanoDrop Technologies, Wilmington, DE, USA) and for integrity by electrophoresis on a 2100 BioAnalyzer (Agilent Technologies, Santa Clara, CA, USA).

### α-Amanitin experiments with LNCaP cells

The prostate carcinoma cell line LNCaP was obtained from ATCC and maintained in RPMI 1640 medium (Invitrogen) supplemented with 10% (vol/vol) fetal calf serum, 3 mM L-glutamine, 100 μg/ml streptomycin and 100 U/ml penicillin, at 37°C and 5% CO_2_. For RNAP II inhibition experiments, 8 × 10^5 ^LNCaP cells were plated in p60 dishes and cultured for 2 days, after which the medium was replaced by fresh medium with or without (mock) 50 μg/ml α-amanitin (Roche, Basel, Switzerland). After 24 h, the cells were washed once with ice-cold phosphate-buffered saline, harvested, pelleted and stored at -80°C. Total RNA was isolated using Qiagen RNeasy kit and treated with DNase I following the 'on-column DNase digestion' protocol (Qiagen). RNA quality was checked as described above. Two biological replicas were processed separately.

### Sample labeling and microarray hybridization procedures

Cy5- and Cy3-labeled cRNA was obtained using 300 ng total RNA as template for amplification of poly(A) RNA by T7-RNA polymerase with the Agilent Low RNA Input Fluorescent Linear Amplification kit. The T7-polymerase amplified cRNA labeling approach advantageously replaces the reverse-transcriptase cDNA labeling used in early microarray experiments, because T7-RNA polymerase labeling of cRNA preserves the strand orientation of the original mRNA template. Reverse-transcriptase labeling can eventually generate a complementary cDNA second strand and cause artifactual labeling of a target with the opposite sense to that of the original message. For LNCaP cell line samples (mock-treated or α-amanitin-treated cells), 500 ng total RNA was used and a control *in vitro *synthesized mRNA (Agilent RNA Spike-In kit) was spiked into the amplification and labeling assay. For kidney tissue samples, the four pools from normal individuals were considered as replicas, and each pair was labeled with either Cy3 or Cy5. Each liver sample pool, prostate tissue sample or LNCaP cell line sample was separately labeled in replicate with Cy3 or Cy5. Hybridization of 750 ng each of Cy3- and Cy5-labeled cRNA was performed with an Agilent *in situ *Hybridization kit-plus, as recommended by the manufacturer, using a total of six 44 k intron-exon expression oligoarrays. Slides were washed and processed according to the Agilent 60-mer Oligo Microarray Processing protocol and scanned on a GenePix 4000B scanner (Molecular Devices, Sunnyvale, CA, USA). Data were extracted from the images with ArrayVision 8.0 (Imaging Research Inc., GE Healthcare, Piscataway, NJ, USA). Cy5- and Cy3-derived intensity data from the same sample were corrected for intensity-dependent dye biases [66] using a Lowess function implemented in the R package [67]. The different experiments with human tissues were normalized by the 40% trimmed mean intensity of all the spots in each slide that were above the mean plus 2 SD intensity of 1,198 negative controls. The experiments with the LNCaP cells (mock- and α-amanitin-treated cells) were normalized by the 40% trimmed mean intensity of 300 control probes from a specific probe set on the array that reports the signals from labeled targets generated from the synthetic spiked-in mRNA.

### Statistical analyses

For tissue-specific expression profiles, the SAM approach was employed using as parameters: multi-class response, 1,000 permutations, K-Nearest Neighbors Imputer, and FDR ≤ 0.002. Analysis of variance (ANOVA), implemented in the SpotFire Decision Site for Functional Genomics (SpotFire Inc., Somerville, MA, USA) with cutoff *p *≤ 0.001 was also used. Gene sets identified by SAM or ANOVA were combined in order to identify a more restricted set of genes that showed statistically significant changes of expression in a tissue by both analyses.

For RNAP II inhibition experiments, the SAM approach was employed, using as parameters: two-class unpaired response, t-statistic, 1,000 permutations, K-Nearest Neighbors Imputer, and FDRs ranging from 0.2% to 2%; a signal-to-noise ratio (SNR) analysis with 10 k permutations (*p *< 0.05) was performed. Gene sets identified by SAM or SNR were combined in order to identify a more restricted set of genes that showed statistically significant changes of expression upon α-amanitin treatment by both analyses.

### GO enrichment analyses

We used BiNGO, the Biological Network Gene Ontology plug-in tool [35] version 1 from the Cytoscape package [68], with a GO database updated as of 17 June 2006. BiNGO analysis does not include eventual duplicate instances of the same Gene_ID in a given selected dataset; only one event is counted for a given Gene_ID. We used the Hypergeometric statistical test with Benjamini and Hochberg's FDR multiple testing correction, choosing a significance level of 0.05. We used as the reference dataset all genes that were present in our 44 k intron-exon expression oligoarray, as follows: for protein-coding genes, we used all Gene_IDs of protein-coding probes in the array; for TIN and PIN RNAs, we used all Gene_IDs to protein-coding genes for which there were TIN or PIN RNA probes in the array mapping to the corresponding genomic loci.

### Accession numbers

Related microarray data are deposited at Gene Expression Omnibus (GEO) under accession numbers [GenBank:GSE5452, GenBank:GSE5453].

## Additional data files

The following additional data are available with the online version of this paper. Additional data file 1 lists sense/antisense transcript pairs with overlapping exons and with no exon overlap (wholly intronic) identified in the RefSeq and mRNA data from GenBank. Additional data file 2 shows abundance of wholly intronic noncoding transcription in RefSeq genes. Additional data file 3 shows the distribution of BLAST bit-score for the second best hit of the 60-mer oligonucleotide probes from the microarray. Additional data file 4 shows that the most highly expressed antisense TIN transcripts map to genes related to regulation of transcription. The table shows the exact *p *values for all significantly enriched GO categories for each of the three tissues studied. Additional data file 5 is a list of 210 probes representing antisense TIN RNAs from 123 Gene IDs of genes related to 'Regulation of transcription'.

Additional data file 6 provides gene ontology analyses with the most highly expressed protein-coding transcripts in three different human tissues. Additional data file 7 lists exact *p *values for all significantly enriched GO categories of genes with up-regulated intronic transcription in the presence of α-amanitin. All exonic protein-coding and intronic non-coding RNAs up-regulated upon alpha-amanitin treatment are also shown. Additional data file 8 lists the tissue signatures of 431 antisense PIN RNAs. Additional data file 9 lists the tissue signatures of 419 antisense TIN RNAs. Additional data file 10 lists the tissue signatures of 567 sense TIN RNAs. Additional data file 11 is a comparison of tissue signatures between antisense PIN RNAs and exons of protein-coding genes. Additional data file 12 is a comparison of tissue signatures between TIN RNAs and exons of protein-coding genes. Additional data file 13 provides the genomic coordinates of all 55,139 TIN RNAs (formatted for UCSC browser track, hg17 assembly version of May 2004). Additional data file 14 provides the genomic coordinates of all 12,592 PIN RNAs (formatted for UCSC browser track, hg17 assembly version of May 2004). Additional data file 15 shows the 44 K platform design. Additional data file 16 provides the Genomic coordinates of all intronic and exonic probes in the custom-designed 44 K intron-exon oligoarray (formatted for UCSC browser track, hg17 assembly version of May 2004).

## Supplementary Material

Additional data file 1 Sense/antisense transcript pairs with overlapping exons and with no exon overlap (wholly intronic) identified in the RefSeq and mRNA data from GenBank Click here for file

Additional data file 2 Abundance of wholly intronic noncoding transcription in RefSeq genes Click here for file

Additional data file 3 Distribution of BLAST bit-score for the second best hit of the 60-mer oligonucleotide probes from the microarray Click here for file

Additional data file 4 The table shows the exact *p *values for all significantly enriched GO categories for each of the three tissues studied Click here for file

Additional data file 5 probes representing antisense TIN RNAs from 123 Gene IDs of genes related to 'Regulation of transcription' Click here for file

Additional data file 6 Gene ontology analyses with the most highly expressed protein-coding transcripts in three different human tissues Click here for file

Additional data file 7 All exonic protein-coding and intronic non-coding RNAs up-regulated upon α-amanitin treatment are also shown Click here for file

Additional data file 8 Tissue signatures of 431 antisense PIN RNAs Click here for file

Additional data file 9 Tissue signatures of 419 antisense TIN RNAs Click here for file

Additional data file 10 Tissue signatures of 567 sense TIN RNAs Click here for file

Additional data file 11 Comparison of tissue signatures between antisense PIN RNAs and exons of protein-coding genes Click here for file

Additional data file 12 Comparison of tissue signatures between TIN RNAs and exons of protein-coding genes Click here for file

Additional data file 13 Formatted for UCSC browser track, hg17 assembly version of May 2004 Click here for file

Additional data file 14 Formatted for UCSC browser track, hg17 assembly version of May 2004 Click here for file

Additional data file 15 Design of the 44 K platform Click here for file

Additional data file 16 Formatted for UCSC browser track, hg17 assembly version of May 2004 Click here for file
