# Supplementary material for: Genome mapping and expression analyses of human intronic noncoding RNAs reveal tissue-specific patterns and enrichment in genes related to regulation of transcription
Source: Genome Biol. 2007 Mar 26;8(3):R43. doi: 10.1186/gb-2007-8-3-r43 (PMC1868932; doi:10.1186/gb-2007-8-3-r43)

Additional data file 2. Abundance of wholly intronic noncoding transcription in RefSeq genes. Distribution of TIN transcripts among the introns of RefSeq sequences with 7, 8, 9 or 10 introns that were selected from GenBank as showing a correlation between the abundance of TIN contigs per intron and the average intron size (in nt). A Pearson correlation analysis and a 95% confidence level of significance were used. Bars indicate the mean intron size per intron (in nt) for this selected set of genes. Triangles indicate the number of TIN contigs per intron for RefSeq genes for the same set.

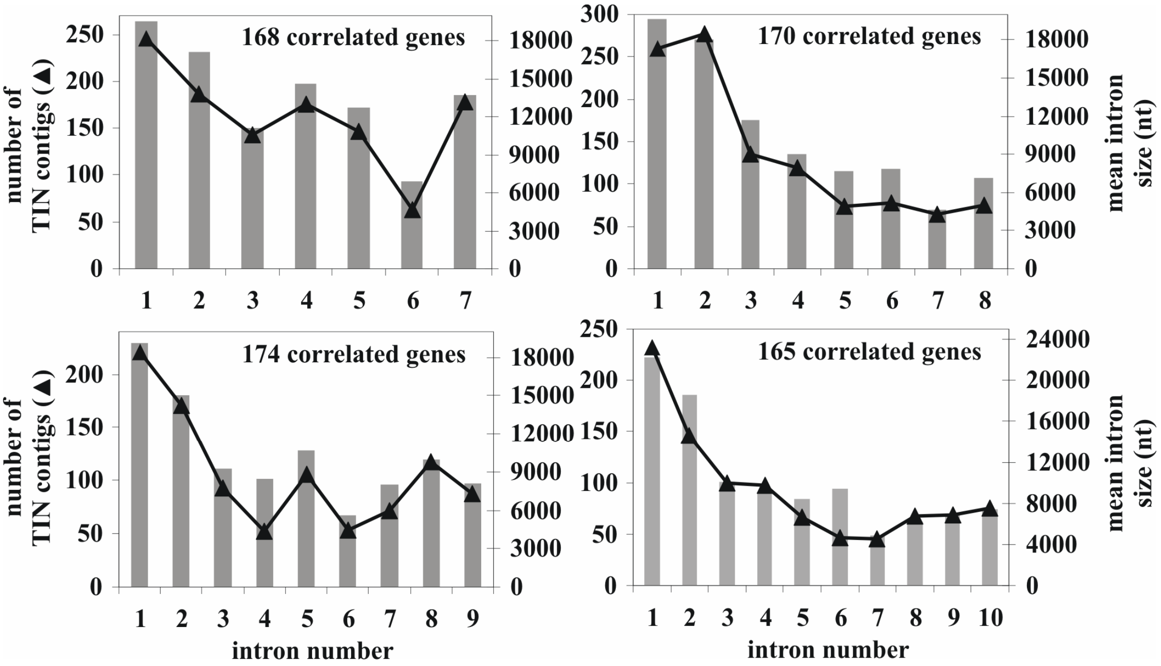

Supplement: Additional data file 2 — Abundance of wholly intronic noncoding transcription in RefSeq genes [file gb-2007-8-3-r43-S2.pdf]
