# Supplementary material for: Genome mapping and expression analyses of human intronic noncoding RNAs reveal tissue-specific patterns and enrichment in genes related to regulation of transcription
Source: Genome Biol. 2007 Mar 26;8(3):R43. doi: 10.1186/gb-2007-8-3-r43 (PMC1868932; doi:10.1186/gb-2007-8-3-r43)

Additional data file 3. Distribution of BLAST bit-score for the second best hit of the 60-mer oligonucleotide probes from the microarray. Each probe sequence was used as query in a BLAST search against all human genome sequences for which there is any evidence of transcription. For each probe, the bit-score result for the second best hit was recorded. Bit-score 42.1 was used as a high-end cutoff for the custom-designed intronic probes selected for our arrays.

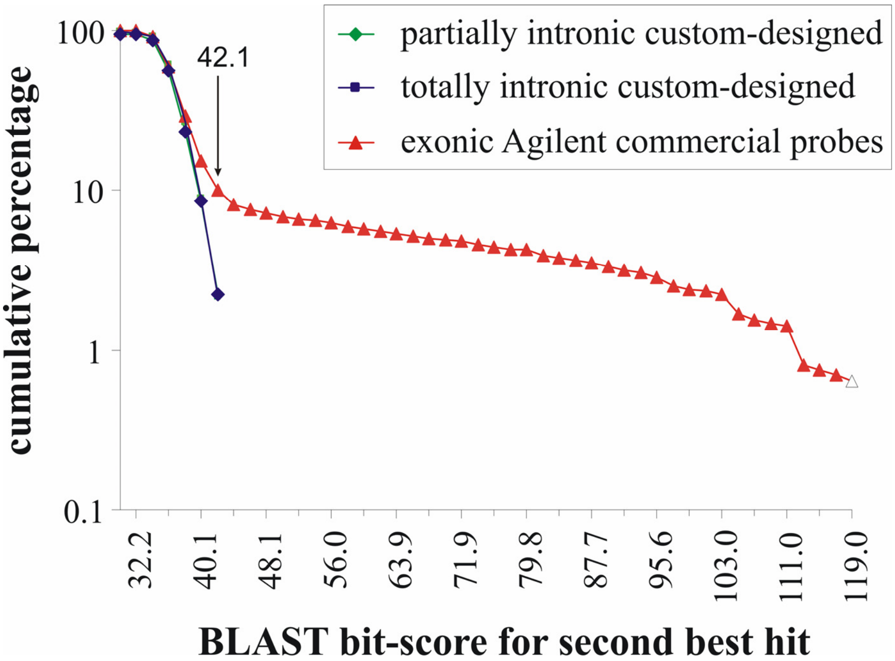

Supplement: Additional data file 3 — Distribution of BLAST bit-score for the second best hit of the 60-mer oligonucleotide probes from the microarray [file gb-2007-8-3-r43-S3.pdf]
