# Supplementary figures and images for: Genome mapping and expression analyses of human intronic noncoding RNAs reveal tissue-specific patterns and enrichment in genes related to regulation of transcription
Source: Genome Biol. 2007 Mar 26;8(3):R43. doi: 10.1186/gb-2007-8-3-r43 (PMC1868932; doi:10.1186/gb-2007-8-3-r43)

**B**

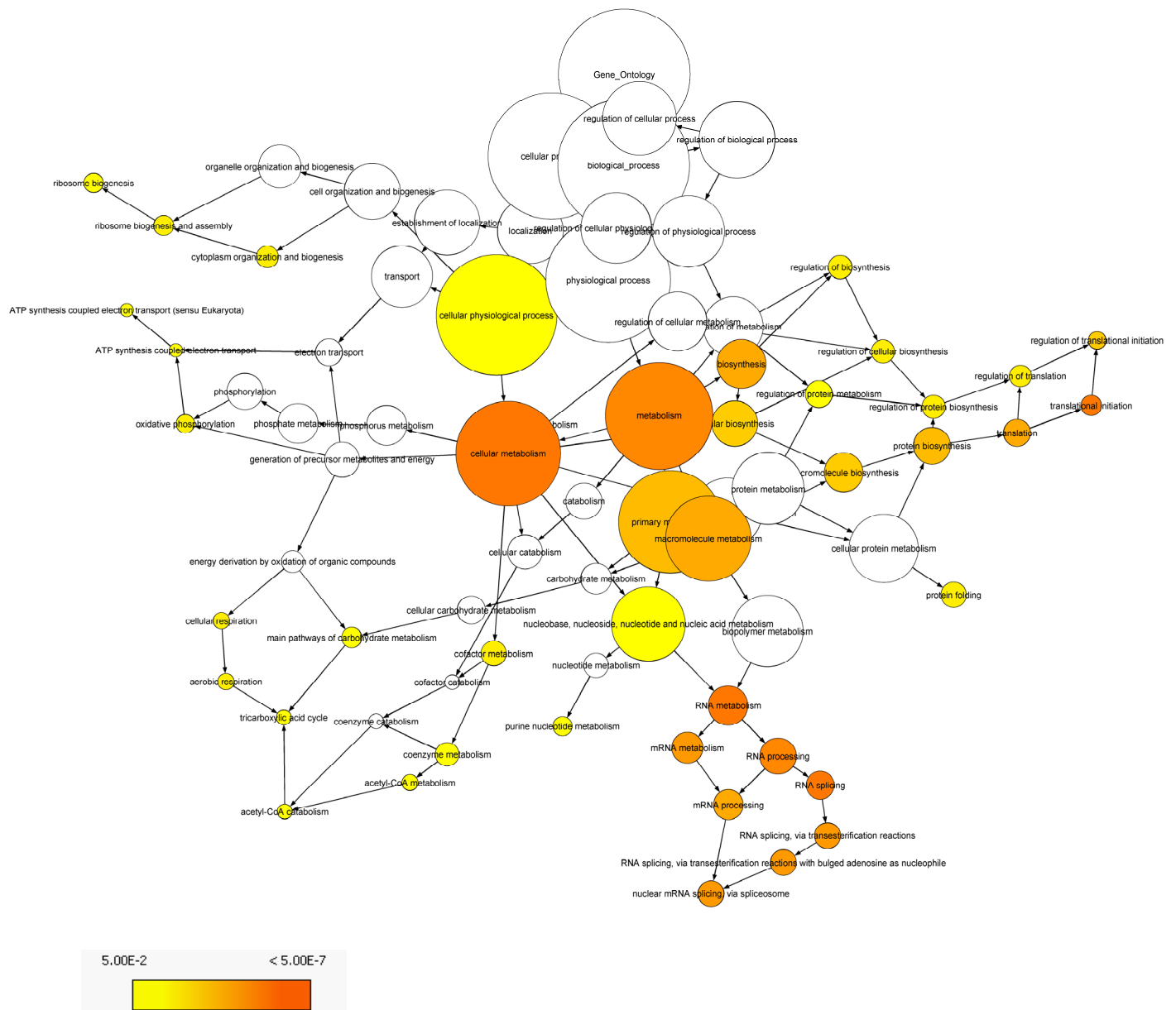

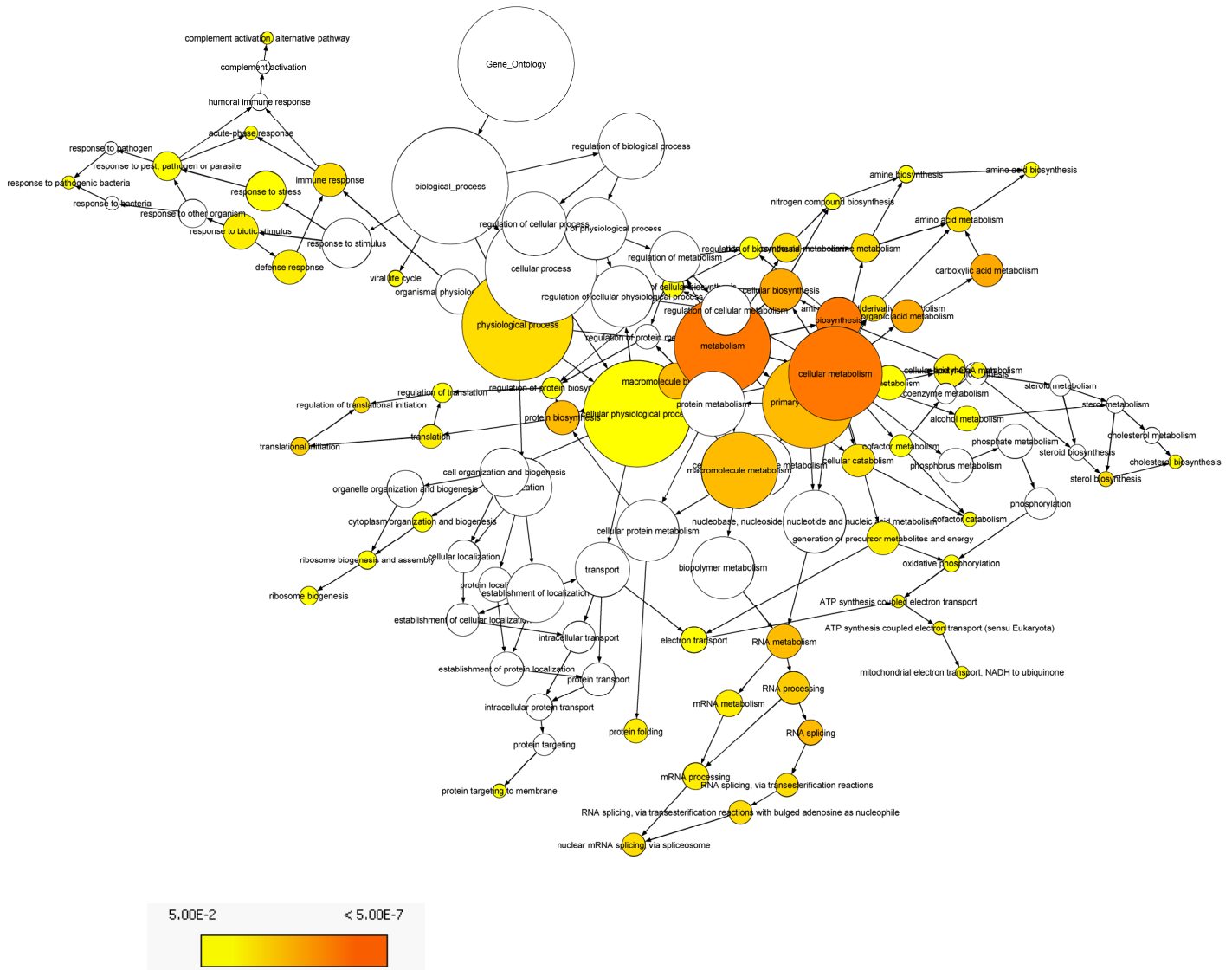

Supplement: Additional data file 6 — Gene ontology analyses with the most highly expressed protein-coding transcripts in three different human tissues [file gb-2007-8-3-r43-S6.pdf]
